# Supplementary material for: The cancer glycocalyx mediates intravascular adhesion and extravasation during metastatic dissemination
Source: Commun Biol. 2021 Feb 26;4:255. doi: 10.1038/s42003-021-01774-2 (PMC7910477; doi:10.1038/s42003-021-01774-2)
Supplement: Supplementary file 2 — Supplementary information [file 42003_2021_1774_MOESM2_ESM.pdf]

SUPPLEMENTARY MATERIAL

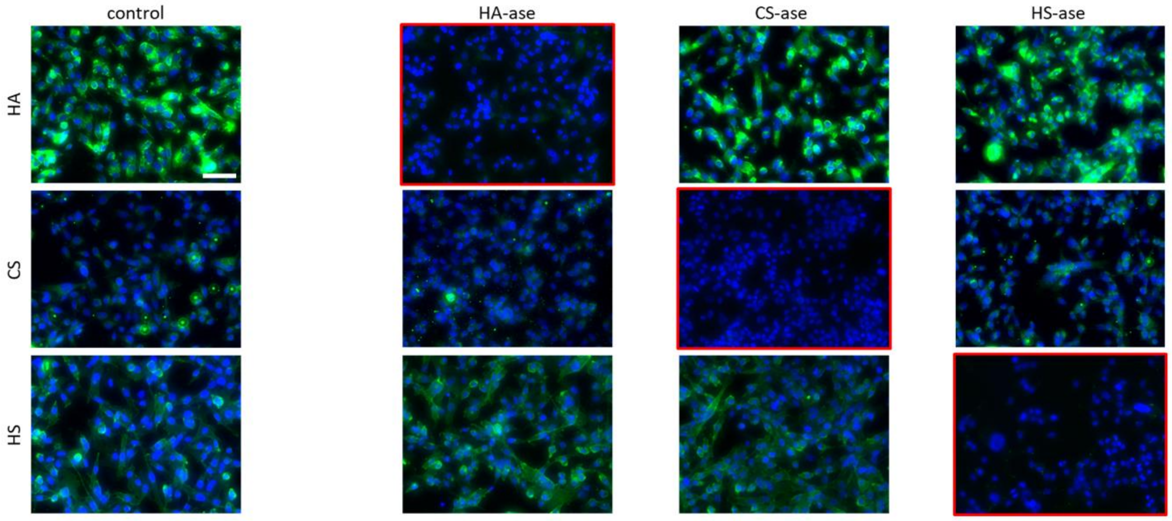

Supplementary Figure 1. Cross-reactivity of the enzymatic treatments used with the GCX components targeted. TCs (MDA-MB-231) in well plates. Blue = DAPI, Green = HA, CS, or HS. The scale bar is 50 µm.

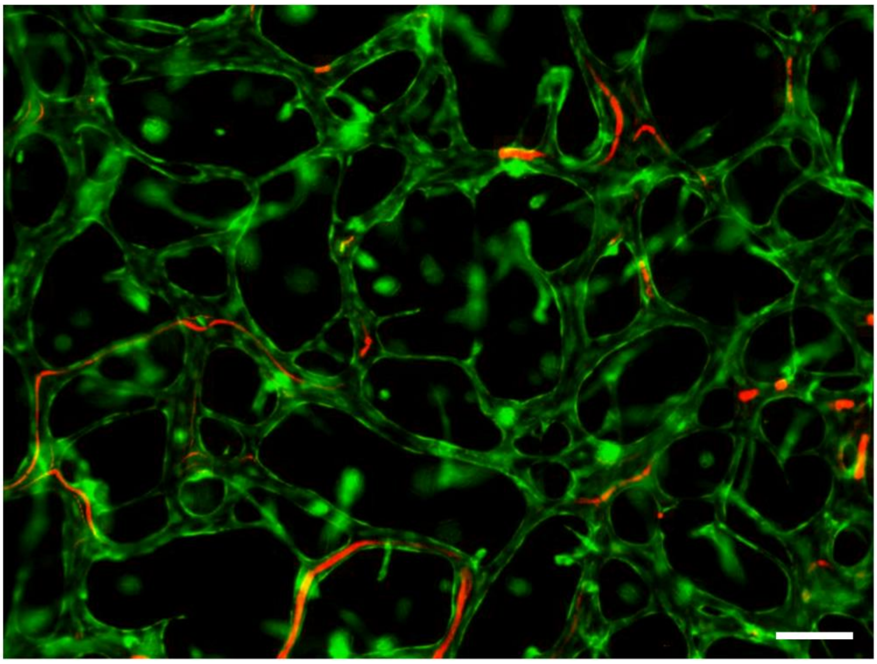

Supplementary Figure 2. Still image of Video 1 showing the paths left by TCs perfused through the MVNs at a constant pressure offset of 400 Pa. Green = ECs (GFP), Red = TCs (RFP). The scale bar is 100 µm.

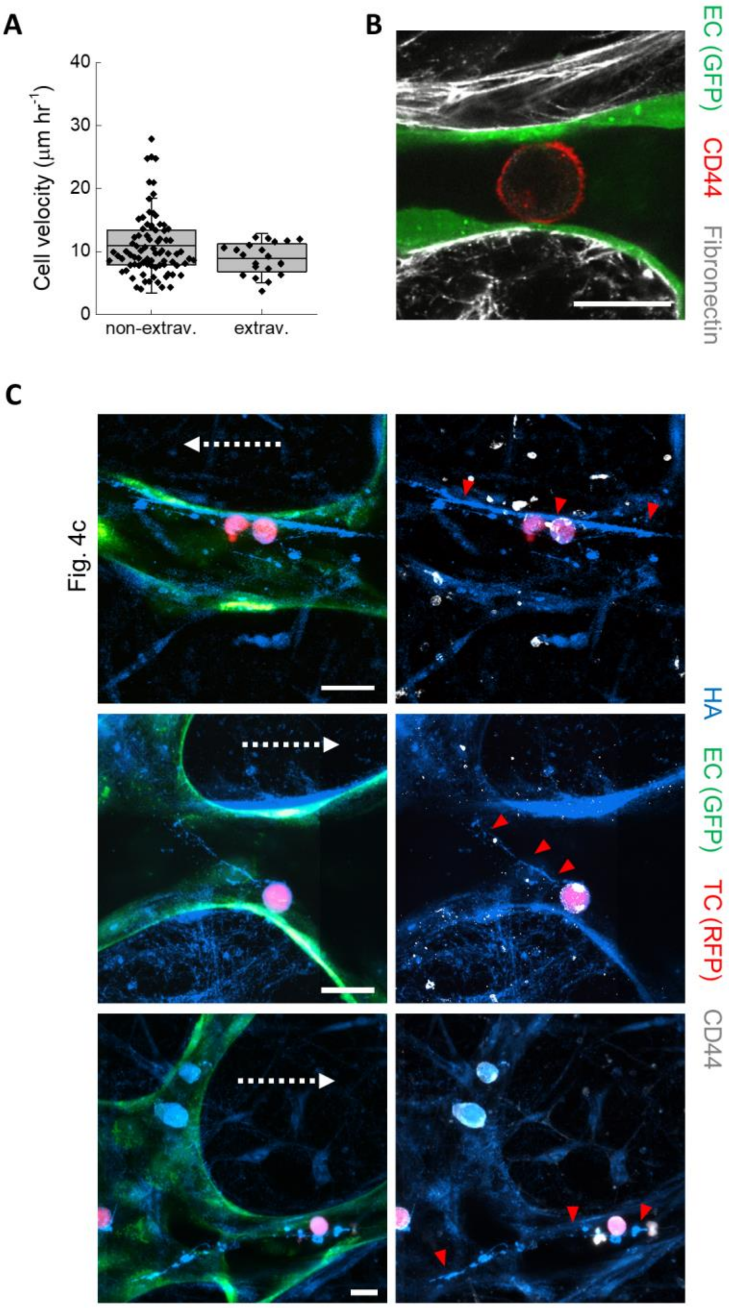

Supplementary Figure 3. (a) Velocity of TCs (MDA-MB-231) within the vascular space of the MVNs as a function of successful extravasation. The data points indicate single cells tracked. The error bars indicate the standard deviation. (b) Confocal imaging of arrested TC in the MVNs showing that binding to fibronectin (here only located in the basement membrane and interstitium) does not take place. (c) Examples of arrested TCs in the MVNs and their deposited HA streaks (red arrows).

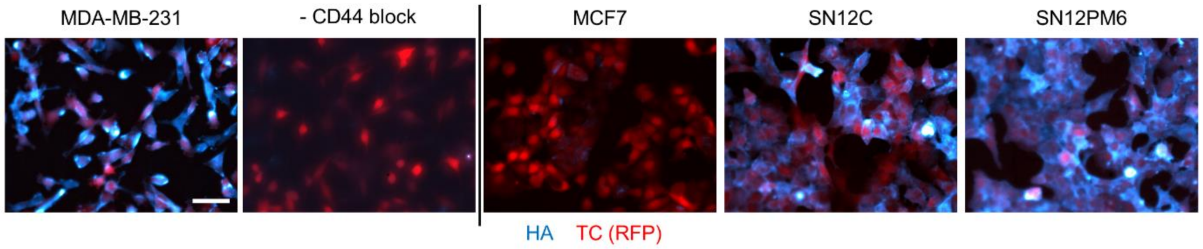

Supplementary Figure 4. Fluorescent imaging of HA expressed by each TC considered, including after treatment of MDA-MB-231 cells with the CD44 blocking antibody.

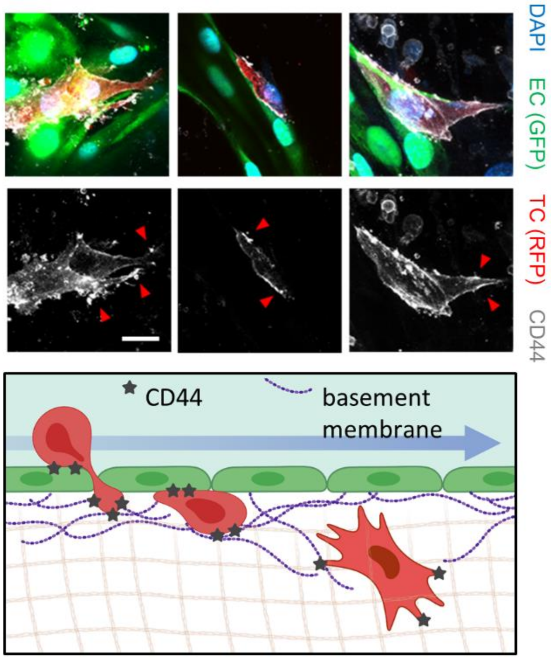

Supplementary Figure 5. Confocal images of fixed TCs undergoing extravasation in the MVNs. The arrows point to CD44 focal points.
