# Supplementary material for: The cancer glycocalyx mediates intravascular adhesion and extravasation during metastatic dissemination
Source: Commun Biol. 2021 Feb 26;4:255. doi: 10.1038/s42003-021-01774-2 (PMC7910477; doi:10.1038/s42003-021-01774-2)
Supplement: Supplementary file 3 — Description of Supplementary Files [file 42003_2021_1774_MOESM3_ESM.pdf]

Description of Additional Supplementary Files

File name: Supplementary Video 1.

Description: Real time imaging of TCs (red, RFP) flowing through the MVNs (green, GFP) under a 400 Pa pressure differential. The scale bar is 150 μm.

File name: Supplementary Video 2.

Description: Time-course imaging of arrested TCs (red) migrating within, and extravasating from the MVNs (green). The time between frames is 30 min.

File name: Supplementary Video 3.

Description: Time-course imaging of CD44 (white)-mediated extravasation of a TC from MVNs (green). The frame time is 30 min. CD44 focal points mediate adhesion to the endothelium and binding to the sub-endothelial ECM.

File name: Supplementary Video 4.

Description: Time-course imaging

File name: Supplementary Data 1.

Description: Data underlying manuscript figures.
