# Supplementary material for: The cancer glycocalyx mediates intravascular adhesion and extravasation during metastatic dissemination
Source: Commun Biol. 2021 Feb 26;4:255. doi: 10.1038/s42003-021-01774-2 (PMC7910477; doi:10.1038/s42003-021-01774-2)
Supplement: Supplementary file 9 — Reporting Summary [file 42003_2021_1774_MOESM9_ESM.pdf]

nature research

Corresponding author(s): Roger D. Kamm, Mark F. Coughlin

Last updated by author(s): Jan 10, 2021

Reporting Summary

Nature Research wishes to improve the reproducibility of the work that we publish. This form provides structure for consistency and transparency in reporting. For further information on Nature Research policies, see our Editorial Policies and the Editorial Policy Checklist.

Statistics

For all statistical analyses, confirm that the following items are present in the figure legend, table legend, main text, or Methods section.

n/a	Confirmed	
☐	☒	The exact sample size (*n*) for each experimental group/condition, given as a discrete number and unit of measurement
☐	☒	A statement on whether measurements were taken from distinct samples or whether the same sample was measured repeatedly
☐	☒	The statistical test(s) used AND whether they are one- or two-sided<br>*Only common tests should be described solely by name; describe more complex techniques in the Methods section.*
☒	☐	A description of all covariates tested
☒	☐	A description of any assumptions or corrections, such as tests of normality and adjustment for multiple comparisons
☐	☒	A full description of the statistical parameters including central tendency (e.g. means) or other basic estimates (e.g. regression coefficient) AND variation (e.g. standard deviation) or associated estimates of uncertainty (e.g. confidence intervals)
☐	☒	For null hypothesis testing, the test statistic (e.g. *F*, *t*, *r*) with confidence intervals, effect sizes, degrees of freedom and *P* value noted<br>*Give P values as exact values whenever suitable.*
☒	☐	For Bayesian analysis, information on the choice of priors and Markov chain Monte Carlo settings
☒	☐	For hierarchical and complex designs, identification of the appropriate level for tests and full reporting of outcomes
☒	☐	Estimates of effect sizes (e.g. Cohen's *d*, Pearson's *r*), indicating how they were calculated

Our web collection on statistics for biologists contains articles on many of the points above.

Software and code

Policy information about availability of computer code

Data collection	No specialized software was used for data collection.
Data analysis	As per the Methods, the ImageJ distribution Fiji was used for image analysis, including measurement of endothelial permeability, tumor cell extravasation, arrest, and migration. The software OriginPro was used for statistical analysis.

For manuscripts utilizing custom algorithms or software that are central to the research but not yet described in published literature, software must be made available to editors and reviewers. We strongly encourage code deposition in a community repository (e.g. GitHub). See the Nature Research guidelines for submitting code & software for further information.

Data

Policy information about availability of data

All manuscripts must include a data availability statement. This statement should provide the following information, where applicable:

- Accession codes, unique identifiers, or web links for publicly available datasets
- A list of figures that have associated raw data
- A description of any restrictions on data availability

The datasets generated during the current study are available from the corresponding authors on reasonable request. All data underlying the graphs presented in the main figures is available as Supplementary Data.

Field-specific reporting

Please select the one below that is the best fit for your research. If you are not sure, read the appropriate sections before making your selection.

[x] Life sciences [ ] Behavioural & social sciences [ ] Ecological, evolutionary & environmental sciences

For a reference copy of the document with all sections, see nature.com/documents/nr-reporting-summary-flat.pdf

Life sciences study design

All studies must disclose on these points even when the disclosure is negative.

Sample size	Sample size was chosen based on previous studies by us and others on the use of microfluidic models for assessment of biological phenomena. As demonstrated by the single data points in each figure, the sample size chosen described well the variability in the measurements.
Data exclusions	For the tumor cell extravasation assay, regions of interest that contained clumps of tumor cells rather than single cells were removed due to abnormally high extravasation efficiency.
Replication	The permeability and tumor extravasation values measured are comparable to those reported by us in the past.
Randomization	Not applicable to this study.
Blinding	Blinding was performed when comparing the cell lines SN12C and SN12PM6. For these cells, arrest and extravasation efficiencies, and CD44 and HA expressions were each measured by the first author without knowledge of the cell line.

Reporting for specific materials, systems and methods

We require information from authors about some types of materials, experimental systems and methods used in many studies. Here, indicate whether each material, system or method listed is relevant to your study. If you are not sure if a list item applies to your research, read the appropriate section before selecting a response.

Materials & experimental systems

n/a	Involved in the study	
[ ]	[x]	Antibodies
[ ]	[x]	Eukaryotic cell lines
[x]	[ ]	Palaeontology and archaeology
[x]	[ ]	Animals and other organisms
[x]	[ ]	Human research participants
[x]	[ ]	Clinical data
[x]	[ ]	Dual use research of concern

Methods

n/a	Involved in the study	
[x]	[ ]	ChIP-seq
[x]	[ ]	Flow cytometry
[x]	[ ]	MRI-based neuroimaging

Antibodies

Antibodies used	As per the Methods, the antibodies used were against E-selectin (BBA16, R&D Systems), P-selectin (701257, ThermoFisher), VCAM-1 (AB134047, Abcam), ICAM-1 (MA5407, ThermoFisher), CD44 (GTX102111, GeneTex), CD31 (ab32457, Abcam), β-actin (926-42210, Li-Cor), ICAM-1 (62133S, Cell Signaling Technologies), ZO-1 (33-9100, ThermoFisher), and HS (370255, Amsbio).
Validation	All antibodies were validated by the manufacturers as described on their web pages.

Eukaryotic cell lines

Policy information about cell lines

Cell line source(s)	MDA-MB-231 and MCF7 cell lines were obtained from ATCC. SN12C and SN12PM6 cells were obtained from the MD Anderson Characterized Core Facility.
Authentication	Authentication of the cell lines was performed by the suppliers.
Mycoplasma contamination	All cell lines in our laboratory tested negative to mycoplasma contamination during periodic testing.
Commonly misidentified lines (See ICLAC register)	None of the lines used in this study are present in the ICLAC register.
